# Homocysteine Intracerebroventricular Injection Induces Apoptosis in the Substantia Nigra Cells and Parkinson's Disease LikeBehavior in Rats

**Published:** 2013

**Authors:** Amin Ataie, Ramin Ataee, Zahra Mansoury, Mohsen Aghajanpour

**Affiliations:** 1 *Cellular and Molecular Biology Research Center (CMBRC), Babol University of Medical Sciences, Babol, Iran.*; 2 *Pharmaceutical Sciences Research Center, Department of Pharmacology and Toxicology Mazandaran University of Medical Sciences, Sari, Iran.*; 3 *Neuroscience research center. Shahid Beheshti University of Medical Sciences, Tehran, Iran. *

**Keywords:** Homocysteine, parkinson disease, locomotor activity, substantia nigra, immunohistochemistry

## Abstract

Parkinson's disease is a degenerative disorder of the central nervous system. The motor symptoms of Parkinson's disease result from the death of dopamine-generating cells in the substantia nigra, a region of the midbrain; the cause of this cell death is unknown. Homocysteine (Hcy) is a non-protein amino acid. It is a homologue of the amino acid cysteine. The elevated levels of homocysteine in plasma have been associated with a number of disease states. Hcy (2 µmol / µl) was injected intracerebroventricular (i.c.v) in rats, five days later, the locomotor activity was measured with open field apparatus, Also apoptosis was investigated in substantia nigra cells by immunohistochemical analysis. Hcy could decrease locomotor activities significantly in rats as well as it could induce apoptosis in substantia nigra cells. These results suggest that Hcy is a neurotoxic metabolite and may induce cell death in some nuclei in the brain.

Parkinson’s disease (PD) is the second most common neurodegenerative disorder after Alzheimer’s disease (AD) (1) Also, it is progressive and leads patients to a debilitating condition and includes as well extensive dopaminergic neuron degeneration in the substantia nigra pars compacta (2) and the other subcortical nuclei with motor and non-motor symptoms. Motor symptoms are discriminated by hypokinesia, rigidity, tremor, and postural imbalance (3) and non-motor symptoms including autonomic dysfunction, neuropsychiatric problems, and sensory and sleep difficulties, which are common. Homocysteine is considered a risk factor for multiple neurological disorders including AD and PD (4, 5, 6). Homocysteine (Hcy); a sulfur containing amino acid derived from the metabolism of methionine, is an independent risk factor for cardiovascular disease (7). The thiol group of Hcy is readily oxidized in plasma and culture medium, resulting in the generation of reactive oxygen species (ROS). Moreover, Hcy has the ability to inhibit the expression of antioxidant enzymes such as glutathione peroxidase (GSH-Px), and super oxide dismutase (SOD) (8). Hcy is an excitatory amino acid, which markedly enhances the vulnerability of neuronal cells to excitotoxic and oxidative injury (8). An elevated plasma level of Hcy (more than 14 μM) is termed Hyper-homocysteinemia (HHCY) (9). 

Furthermore, it has been suggested that the involved pathological mechanisms of Hcy toxicity are apoptosis, neuronal death, oxidative stress, over activation of glutamate receptors, mitochondrial dysfunctions, and activation of Caspase for all of neurodegenerative diseases (10). In spite of many researches in this area, the molecular mechanism of homocysteine-induced neurotoxicity has not been completely established at present.

## Materials and Methods

**Drugs and Biochemical reagents**

D-L-Homocysteine was purchased from Sigma-Aldrich, Germany. Ketamine and xylazine were obtained from ALFASAN Co, Netherlands. Hcy powder was dissolved in hydrochloric acid (1 M) and diluted with PBS (Sigma-Aldrich). The pH of the solution was adjusted at 7.4 by the addition of 0.1 N NaOH. The solutions of Hcy were prepared freshly at a concentration of 2 μmol. The Hcy effective dose (2 μmol/μl) was obtained. (11).

**Animals**

Adult male Wistar rats were taken from the animal house of Babol University of Medical Sciences, Iran weighing between 200 and 250 g. The animals were housed at 22°C in a controlled environment with a 12:12- h light/dark cycle and were given access to standard laboratory food and water. All experiments were carried out in accordance with the National Institutes of Health guidelines 13 and were approved by the Research and Ethics Committee of Babol University of Medical Sciences. We used animal groups with six animals per group. The animals of the control group received PBS with intracerebroventricular injection, the test group received Hcy (2 µmol/µl) i.c.v. Immunohistochemical and behavioral analyses were performed five days after Hcy injection in rats.

**Intracerebroventricular (i.c.v.) Injection**

For i.c.v. drug administration, the rats were anesthetized using ketamine (10 mg/kg) and placed in a stereotaxic apparatus. Permanent 23 gauge stainless steel guide cannula were positioned in the lateral ventricle based on stereotaxic coordinates taken from Paxinos and Watson atlas of rat brain (12) which were as follows: 1 mm posterior to the bregma, 1.6 mm lateral to midline, and 3.6 mm ventral to the surface of the skull. The cannula was fixed using dental cement, and two stainless steel screws were placed into the skull. The rats were allowed to recover 1 week post surgery before performing the experiment. Drugs were injected into the lateral ventricle 5 mm from the surface of the cranium through a polyethylene tube (PE-20) which was attached to a 5-μl Hamilton syringe. Behavioral and histo chemical analyses were performed five days after Hcy injection in rats.

**Measurement of Locomotors Activities**

The effects of Hcy on the rats’ behavior were studied by an open-field apparatus. All experiments were carried out 5 days after Hcy (intracerebroventricular) injection in rats. The measurement was started 3 min after the placement of animals into the monitor in a quiet isolated place with a dim light. After the rats were injected (i.c.v.) with Hcy once a day, they were placed in the locomotors activity monitor (Ethovision-XT; Noldus, Netherlands). The changes in motor activity of the animals were measured. Total distance and velocity were determined. The locomotors activities were determined for 40 min in 6^th^ day, 5 days after Hcy i.c.v injection in rat. (11).

**Brain Histopathological Analysis**

At the end of the behavioral experiments, the rats were deeply anesthetized with a high dose of ketamine (150 mg/kg) and perfused through the ascending aorta with 50–100 ml of 0.9% saline followed by 100–200 ml of fixative solution containing 4% para formaldehyde in 0.1 M phosphate buffer (PB, pH 7.4) followed by 100 ml of 0.1 M PB containing 10% sucrose. Following perfusion, the brains were removed from the skull;

The blocks of forebrain and brainstem were prepared, and after the final steps of preparation (30% sucrose for 2–3 days), sections were cut at a thickness of 50 μm on a freezing microtome (Leica, Germany) and collected in PB (0.1 M). Every second, a section was Nissl stained with 0.1% Bcl-2 (Sigma). The tissue sections were deparaffinized in xylene. The slides were stained with 0.1% cresyl violet according to the procedure in Wilson and Gamble (13) and viewed under a light microscope (Labomed, USA) for the structure and morphology of the cells. Microscopic images were obtained by a CCD camera and DigiPro software. Immunohistochemistry for Bax and Bcl-2 was carried out on formalin-fixed, paraffin-embedded sections according to the manufacturer’s instructions provided for each antibody. Sections were deparaffinized and rehydrated. Antigen retrieval was executed by microwaving in citrate buffer (pH 6) for 1 and 2×5 min for Bax and Bcl-2, respectively. The sections were quenched with 3% hydrogen peroxide (H2O2) in absolute methanol and blocked with 10% normal goat serum (NGS) + 1% bovine serum albumin in PBS for Bax and Bcl-2 and with 5% NGS. Primary antibodies were applied overnight at 4°C. These were either Bax rabbit polyclonal antibody (abcam, 1/100) or Bcl-2 rabbit polyclonal antibody (abcam, 1/100). The sections were washed and then incubated with a ready-to-use anti-rabbit secondary antibody from Dako (EnVision Plus®), and color reaction was developed using diamino benzidine as the chromagen. The slides were then counter-stained with hematoxylin, dehydrated using graded alcohols and xylene, and mounted with Permount mounting medium (Entellan®, MERCK). Sections used as negative controls were incubated with the primary antibody diluents and PBS, instead of the primary antiserum.

**Statistics**

The Bax, Bcl-2, the ratio of Bax to Bcl-2 immunostaining, and Nissl-stained neurons of the SNC were determined at each control and homocysteine groups by using the mean scales of Bax and Bcl-2 in the same animal. All of the data are presented as means ± standard error of the mean (S.E.M.) and analyzed by one-way analysis of variance (ANOVA) as appropriate. A p-value of <0.05 was considered statistically significant.

## Results

**Locomotor Activity**

**Fig 1:**
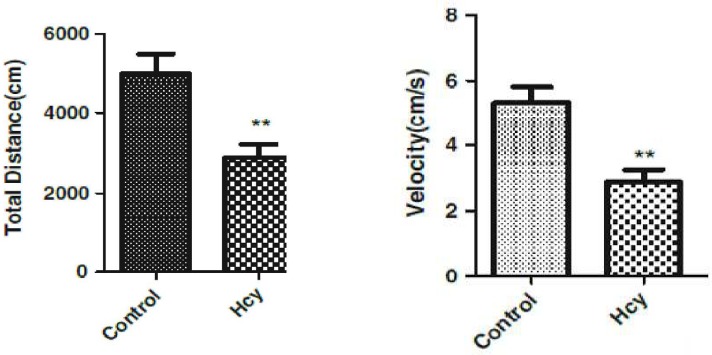
The effect of Hcy (2 micromol) on locomotor activities in rat (total distant , velocity), Data are mean±SEM values (n=6).‏‏P<.001

I.c.v. treatment of homocysteine (2 μmol) decrease the locomotor activity (total distance and velocity) compared to control group (F(1, 10) = 517.5, p<0.001) for total distance and (F(1, 10)=129.5, p<0.001) for velocity (Fig. 1).

**Bax/Bcl-2**

**Fig 2:**
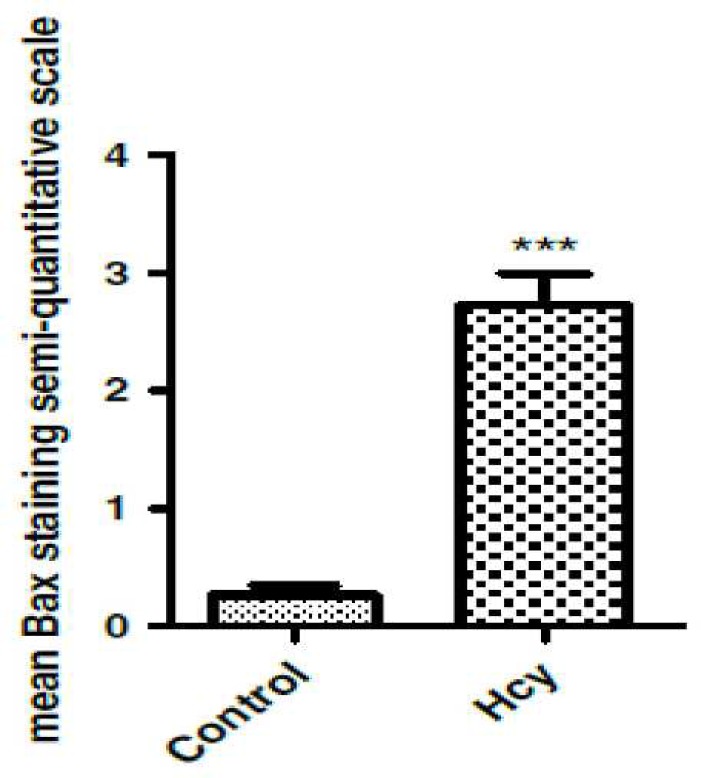
The effect of Hcy on Bax expression in SN cells in the rat brain. Data are mean ± SEM values (n=6). ‏‏P<.001

**Fig 3:**
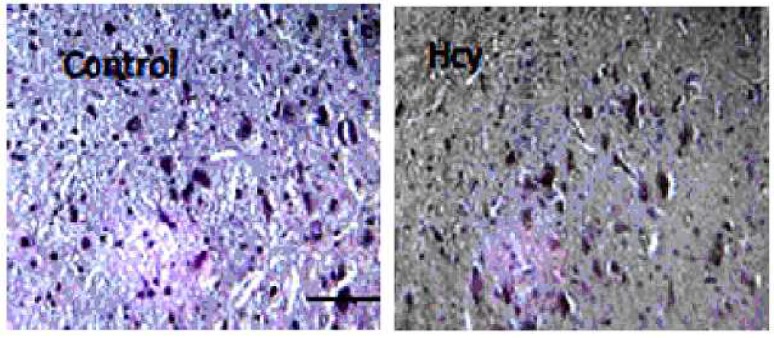
Histologocal analysis of SN cells in the rat brain after Hcy i.c.v injection. Left: control group Right: Hcy group

**Fig 4:**
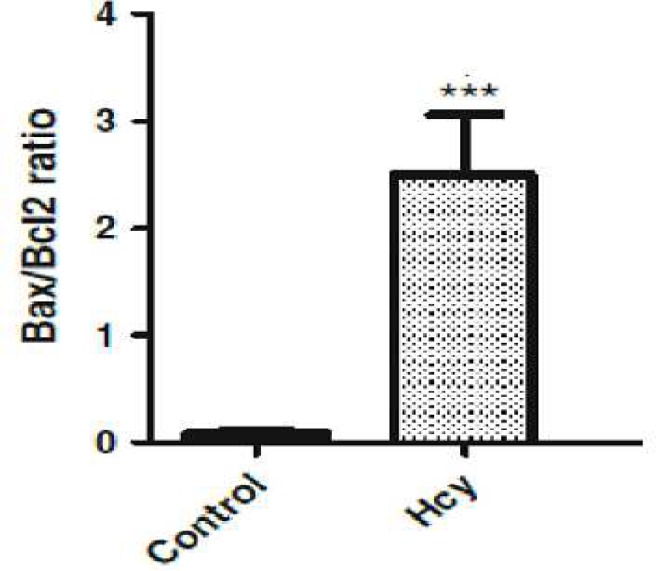
Effect of Hcy on apoptoic biomarkers Bax/Bcl-2 ratio in SN cells in rat brain. Data are mean±SEM values (n=6).‏‏ P<.001

To determine whether homocysteine leads to changes in Bcl-2 family protein levels in the rat brain, we examined the Bcl-2 and Bax protein immunostaining. As shown in fig. 2, Bax protein was detected in control animals and in homocysteine animals; Bax immunostaining significantly increased in control groups (Fig. 2, 3), (F (1, 10) = 1525.5, p<0.001). In contrast with Bax, Bcl-2 staining showed a vigorous expression of this anti apoptotic protein in control group, and this refers to the constitutive expression of Bcl-2 protein in normal conditions In fig. 4, the Bax/Bcl-2 ratio was calculated for SN tissue as explained above. The results showed that in control group, this ratio is lower than homocysteine group which were significantly increased (F (1, 10) = 97.93, p<0.001).

## Discussion

The aim of this study was to investigate the neurotoxic effect of Hcy in SN cells in the rat brain, also the behavioral effect of Hcy injection was investigated in rat. Behavioral results indicated that administration of homocysteine (2 µmol/µl) significantly decreased locomotor activity (total distance and velocity) in comparison with the control groups.

This result is consistent with that of Lee et al. who reported a decrease in the level of loco-motor activity after acute homocysteine injection (11).

Literature data indicate that Hcy is toxic to neuronal cells (14). Moreover, hyperhomo-cysteinemia has been implicated in neuronal plasticity and neurodegenerative disorders in human study (5). The concentration of Hcy in the brain and cerebrospinal fluid is elevated in several neurological diseases in human and experimental animals (5, 15). Numerous studies have reported that homocysteine is elevated in Levodopa therapy for PD patients and suggested a substantial role of homocysteine in causing various neurotoxic effects (16).

In the present study, we investigated the molecular response of the rat brain with immuno-histochemical methods. The results showed that the expression of apoptosis regulatory proteins Bax and Bcl-2 would be altered by homocysteine which elevated the Bax/Bcl-2 ratio in favor of apoptosis. Apoptosis is a morphologically and biochemically well characterized form of programmed cell death to remove the unnecessary or damaged cells in various situations (17). Apoptosis leads to cell death and differs from necrosis by distinct morphologic and biochemical features (18).

A key factor in determining cell death or survival following apoptotic signals is the relative expression of Bax and Bcl-2 proteins. The interactions between these pro-apoptotic and anti-apoptotic proteins regulate the release of cytochrome c and the propagation of apoptotic cascade (19). The role of these apoptotic proteins in adjusting the number of neural precursors and post mitotic neurons during the development of nervous system has been established (20).

The results of the present study showed that Hcy was neurotoxic for rats. Histopathological results revealed that 5 days after Hcy (i.c.v.) injection, Bax level was significantly increased while Bcl-2 level was dramatically decreased in the substantia nigra in comparison to the vehicle and control groups (Figs. 2 and 3). It has been reported that hyperhomocysteinemia causes increase in pro-apoptotic Bax levels and decrease in anti-apoptotic Bcl-2 levels in the rat brain (21). 

Our results suggested that Hcy might induce apoptosis and cell death in rat brain. Hcy may induce oxidative stress and produce ROS that attack all biological macromolecules (e.g. proteins, DNA and lipids). It is suggested that Hcy may be a risk factor for AD and PD.

## References

[1] Hoehn MM (1987). Parkinson's disease: progression and mortality. Adv Neurol.

[2] Lotharius J, Brundin P (2002). Pathogenesis of Parkinson's disease: dopamine, vesicles and alpha-synuclein. Nat Rev Neurosci.

[3] Gomez-Tortosa E, Newell K, Irizarry MC (1999). Clinical and quantitative pathologic correlates of dementia with Lewy bodies. Neurology.

[4] Gottfries CG, Lehmann W, Regland B (1998). Early diagnosis of cognitive impairment in the elderly with the focus on Alzheimer's disease. J Neural Transm.

[5] Mattson MP, Shea TB (2003). Folate and homocysteine metabolism in neural plasticity and neurodegenerative disorders. Trends Neurosci.

[6] Reutens S, Sachdev P (2002). Homocysteine in neuropsychiatric disorders of the elderly. Int J Geriatr Psychiatry.

[7] Clarke R, Daly L, Robinson K (1991). Hyperhomocysteinemia: an independent risk factor for vascular disease. N Engl J Med.

[8] Hankey GJ, Eikelboom JW (1999). Homocysteine and vascular disease. Lancet.

[9] Seshadri S, Beiser A, Selhub J (2002). Plasma homocysteine as a risk factor for dementia and Alzheimer's disease. New Engl J Med.

[10] Mattson MP, Duan W (1999). "Apoptotic" biochemical cascades in synaptic compartments: roles in adaptive plasticity and neurodegenerative disorders. J Neurosci Res.

[11] Lee ES, Chen H, Soliman KF (2005). Effects of homocysteine on the dopaminergic system and behavior in rodents. Neurotoxicology.

[12] Paxinos G, Watson C (2005). The rat brain in stereotaxic coordinates.

[13] Wilson I, Gamble M, Bancroft JD, Gamble M (2002). The hematoxylin and eosin. Theory and practice of histological techniques.

[14] Lipton SA, Kim WK, Choi YB (1997). Neurotoxicity associated with dual actions of homocysteine at the N-methyl-D-aspartate receptor. Proc Natl Acad Sci.

[15] Streck EL, Bavaresco CS, Netto CA (2004). Chronic hyperhomocysteinemia provokes a memory deficit in rats in the Morris water maze task. Behav Brain Res.

[16] Muller T, Woitalla D, Fowler B (2002). 3-OMD and homocysteine plasma levels in parkinsonian patients. J Neural Transm.

[17] Thompson CB (1995). Apoptosis in the Pathogenesis and Treatment of Disease. Science.

[18] Orrenius S, Gogvadze V, Zhivotovsky B (2007). Mitochondrial oxidative stress: implications for cell death. Annu Rev Pharmacol Toxicol.

[19] Deveraux QL, Schendel SL, Reed JC (2001). Antiapoptotic proteins.The bcl-2 and inhibitor of apoptosis protein families. Cardiol Clin.

[20] Krajewska M, Zapata JM, Meinhold-Heerlein I (2002). Expression of Bcl-2 family member Bid in normal and malignant tissues. Neoplasia.

[21] Baydas G, Reiter RJ, Akbulut M (2005). Melatonin inhibits neural apoptosis induced by homocysteine in hippocampus of rats via inhibition of cytochrome c translocation and caspase-3 activation and by regulating pro- and anti-apoptotic protein levels. Neuroscience.

